# QUADRIVEN: A Framework for Qualitative Taxi Demand Prediction Based on Time-Variant Online Social Network Data Analysis

**DOI:** 10.3390/s19224882

**Published:** 2019-11-08

**Authors:** Fernando Terroso-Saenz, Andres Muñoz, José M. Cecilia

**Affiliations:** Polytechnic School, Universidad Católica de Murcia (UCAM), 30107 Murcia, Spain; amunoz@ucam.edu (A.M.); jmcecilia@ucam.edu (J.M.C.)

**Keywords:** taxi demand, online social networks, machine learning, air pollution, smart cities, social media analysis

## Abstract

Road traffic pollution is one of the key factors affecting urban air quality. There is a consensus in the community that the efficient use of public transport is the most effective solution. In that sense, much effort has been made in the data mining discipline to come up with solutions able to anticipate taxi demands in a city. This helps to optimize the trips made by such an important urban means of transport. However, most of the existing solutions in the literature define the taxi demand prediction as a regression problem based on historical taxi records. This causes serious limitations with respect to the required data to operate and the interpretability of the prediction outcome. In this paper, we introduce QUADRIVEN (QUalitative tAxi Demand pRediction based on tIme-Variant onlinE social Network data analysis), a novel approach to deal with the taxi demand prediction problem based on human-generated data widely available on online social networks. The result of the prediction is defined on the basis of categorical labels that allow obtaining a semantically-enriched output. Finally, this proposal was tested with different models in a large urban area, showing quite promising results with an F1 score above 0.8.

## 1. Introduction

Modern cities are becoming complex and large human environments due to the the endless transference of the population from rural to urban areas. As a matter of fact, the United Nations (UN) claims that 68% of the world population will live in urban areas by 2050 (https://www.un.org/development/desa/en/news/population/2018-revision-of-world-urbanization-prospects.html). This endless growth of urban zones poses several challenges for public administrations and stakeholders. One of these challenges is the increase of the road traffic in cities and, thus, of the levels of air pollution, which is actually causing serious health disorders for city dwellers, regardless of their age, gender, or any other factor [1]. Therefore, the governing authorities of modern societies are called upon to seek more efficient public mobility services, as this is the only solution for cities to be truly sustainable.

The taxi service is an important transportation mode in urban areas. Unlike other ridesharing services like Uber (https://www.uber.com), where users hire a ride in advance via Internet applications, taxicabs are usually requested by pedestrians in a more spontaneous manner, which makes taxi behavior much more unpredictable. Several solutions have been proposed from many different disciplines so as to improve the quality of service and the efficiency of urban taxi rides [2,3,4]. In that sense, a foremost course of action within the mobility data mining field has focused on predicting the taxi demand in different areas within a city [5,6,7]. This way, it is possible to inform taxi operators in advance and minimize the amount of time that these vehicles are empty. Although many different approaches have been proposed for taxi demand prediction, it is possible to observe certain limitations:

1. Most proposals address the problem of predicting taxi demand as a regression problem. Thus, they provide prediction outcomes in a quantitative manner (e.g., the future sheer number of pick-ups at a certain area of the city). However, this type of information might not be semantically meaningful in certain scenarios, as it may not refer to a certain contextual situation.
2. Some proposals focus on anticipating taxi demand peaks in areas where the number of taxi pick-ups is expected to be much higher than in a normal situation. Nevertheless, there is a scarcity of proposals able to report a drop in the demand in spite of the fact that this information may be very valuable for operators as well [8,9].
3. Current solutions usually rely on the data generated by the taxi service itself (e.g., GPS traces, pick-up and drop-off details, etc.) to build up the prediction models. This highly limits the scalability of the solutions, as they can only operate in cities with taxi services capable of generating and capturing the data required by the models.

**Figure 1:**
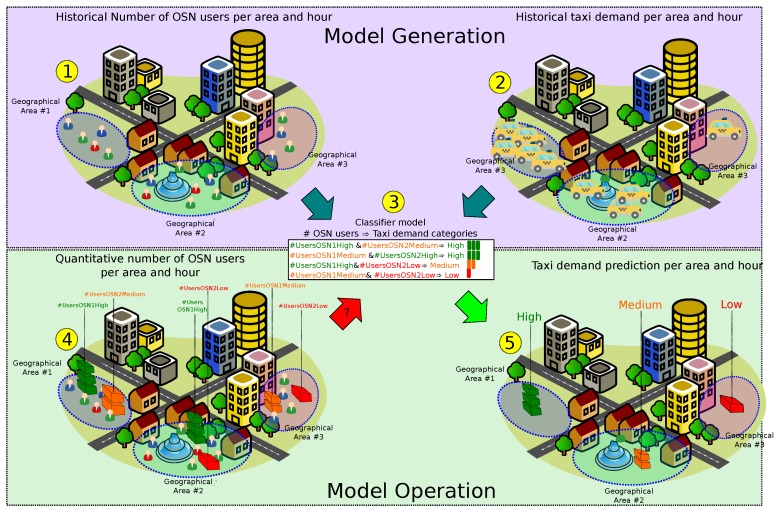
QUADRIVEN (QUalitative tAxi Demand pRediction based on tIme-Variant onlinE social Network data analysis) overview. In Step 1, the sheer number of active OSN users is extracted from two Online Social Network (OSN) platforms (OSN1 and OSN2) in Areas 1, 2, and 3. Similarly, the actual taxi demand in the same areas and time period is extracted in Step 2. In Step 3, a classifier is developed based on the association between the number of users in an area and expected taxi demand in such an area in the short term. In Step 4, the classifier is fed with the number of current active users in the target regions during a time interval. Finally, in Step 5, the predicted taxi demand is generated as categorical data. Notice that the information here is simplified for illustration purpose.

With all of these in mind, we introduce QUADRIVEN, a framework for QUalitative tAxi Demand pRediction based on tIme-Variant onlinE social Network data analysis. Unlike previous proposals, our approach considers the taxi demand prediction as a classification problem instead of a regression one (see Figure 1). This way, QUADRIVEN is able to provide a taxi demand prediction in a qualitative manner, avoiding the aforementioned lack of meaning of quantitative solutions. In that sense, it is true that quantitative outcomes can be converted into categorical data by means of a mapping process. However, this includes an extra level of indexation in the solution pipeline. On the contrary, our approach focuses on training a classifier able to generate this categorical data directly, avoiding this extra indexation level. This training makes the mapping between the input data and the final label very accurate. Moreover, our approach is able to detect both peaks and drops in the taxi demand service in certain urban areas. As discussed above, the prediction of low demand is also important in these scenarios despite being less explored in the literature.

Regarding the target data to make such predictions, QUADRIVEN relies on the fact that Online Social Networks (OSNs) have proven to be suitable proxies to capture human mobility within a city [10]. Hence, we made use of multiple OSN geo-tagged feeds to give insight into future taxi demand behaviors, as is shown in Figure 1. Due to the worldwide deployment of OSN platforms, our proposal can operate in a city without depending on the information available from taxi operators. These operators might be somewhat reluctant to share such sensitive information in business terms with third parties. Note that in this paper, we focus on the aggregation of the geolocation data provided by the OSNs, regardless of the reason for sharing those data. The analysis of the content within the post or the study of user mobility patterns is outside the scope of this paper.

**Figure 2:**
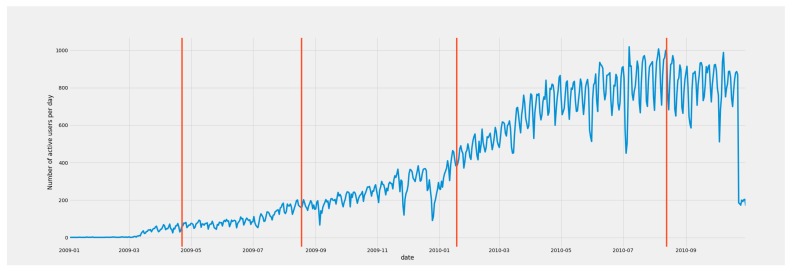
Evolution of the number of active users per day on Foursquare in a two-year period in Manhattan (New York City). Vertical red lines represent breakout points in the time series calculated with the E-Divisive with Median (EDM) algorithm [11].

A paramount feature of our approach is that it smoothly integrates the time-variant nature of OSN feeds in the prediction loop. As an example, Figure 2 depicts the number of users publishing at least one daily check-ins on the OSN Foursquare, in a two-year period in Manhattan (New York City, United States of America). As can be seen, this number of active users remarkably increased during the whole time period, probably due to a popularity increase of the platform. Nonetheless, existing solutions that deal with OSN data do not consider such fluctuation in their pipeline, as they assume a steady behavior of the platform.

More specifically, QUADRIVEN takes as input the number of active OSN users in different areas of the city in a categorical manner (e.g., high, low, medium). Then, a classifying model maps such categories of active users to particular levels of taxi demand for a particular geographic area and time of day. The contributions to the state-of-the-art of the present solution are twofold. Firstly, a novel qualitative approach to predict taxi demand is introduced. This allows reporting about not only peaks, but also meaningful drops of the taxi demand in certain areas in particular time periods. Secondly, it uses different OSN feeds to generate the prediction outcome by considering the inherent variability of such sources. This way, the method adapts itself to changes in the popularity of an OSN feed, providing a more robust solution.

The rest of the paper is structured as follows. Section 2 is devoted to describing in detail the logic structure and the processing stages of the proposed system. Then, Section 3 discusses the main results of the performed experiments. Next, Section 4 provides an overview about taxi demand prediction based on social sensing. Finally, the main conclusions and the future work are summed up in Section 5.

## 2. The QUADRIVEN Framework

**Figure 3:**
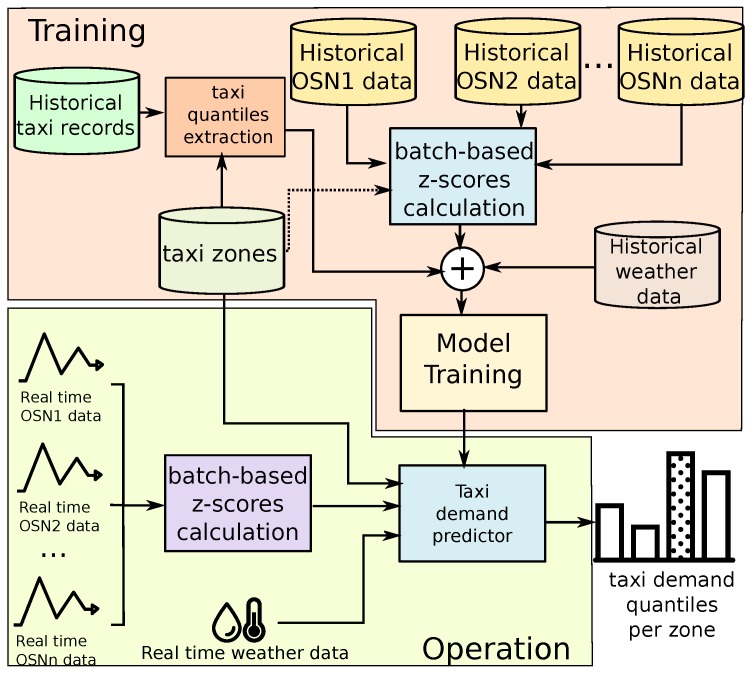
Architecture of QUADRIVEN. In the training stage, historical data from *n* different OSNs are used. From these data, the z-scores associated with the number of active users per taxi zone are calculated following a batch processing. Those scores along with historical weather data are used to compose the independent variables of the training dataset. The dependent variable (label) is generated by extracting the quantile ranges of the taxi demand per taxi zone. Once the model has been trained, it takes the z-scores of the active users considering the last *d* days along with the current weather conditions to compose the qualitative taxi demand prediction per hour and taxi zone.

This section introduces the QUADRIVEN framework in detail. In that sense, we concentrated on Manhattan, one of the five boroughs of New York City (NYC), as the target urban area. Figure 3 depicts the generation and the operational stage of the solution. In what follows, the details of each stage are presented.

### 2.1. Prediction Problem Statement

The prediction problem that the present work deals with can be formulated as follows:

Given an hour h∈〈0,23〉⊂N, an urban region *r*, and the number of active users on *n* different OSN platforms in *r* during the last *t* hours (h−t,h], $U^{rh}=\langle u_{1}^{rh},u_{2}^{rh},\ldots ,u_{n}^{rh}\rangle$, find the taxi demand level in *r* at hour h+1, $tl^{r(h+1)}\in \mathrm{TL}$, where $\mathrm{TL}=\langle tl_{1},tl_{2},..,tl_{k}\rangle$ is an ordered list of *k* categorical taxi demand levels.

Hence, the higher the predicted level $tl_{i}$, the higher the number of estimated taxi users in *r* at hour h+1. The rationale of our approach is that the higher the number of OSN users uploading content in a particular area of a city during a time period, the higher the human activity in such spatio-temporal space and, thus, the higher expectation of people demanding taxi trips.

### 2.2. Data Description

This section explains the different required data in QUADRIVEN for solving the taxi demand prediction problem.

#### 2.2.1. Region Partitioning

QUADRIVEN requires the spatial partition of the target city into different regions $R=\langle r_{1},r_{2},\ldots ,r_{m}\rangle$. This way, the system will provide a different demand estimation for each region $r_{i}\in R$.

A commonly accepted approach has been the definition of these regions as squared cells obtained from a gridded spatial tessellation of the city [6,12,13]. However, these cells might suffer from a lack of meaning as they do not really represent the distribution of the city from a human point of view.

**Figure 4:**
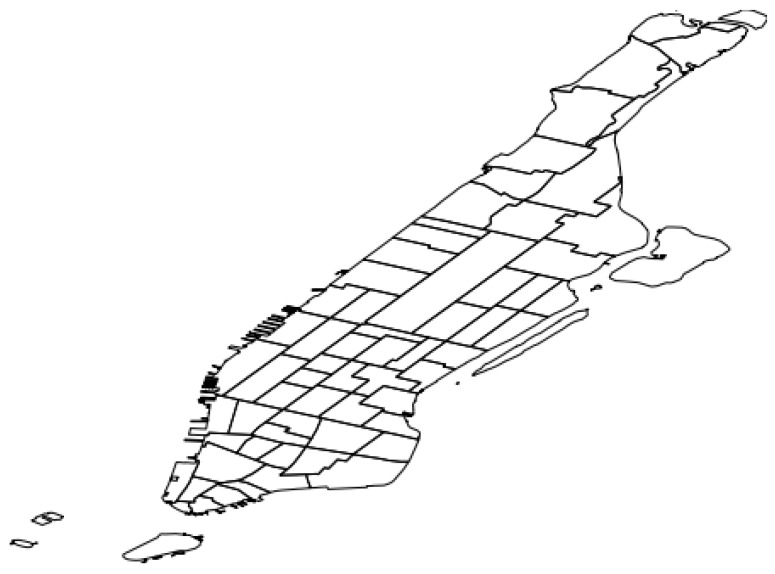
Manhattan taxi zones. Each polygon represents a particular zone.

For that reason, QUADRIVEN relies on the pre-defined region partitioning provided by the official NYC taxi zones (https://data.cityofnewyork.us/Transportation/NYC-Taxi-Zones/d3c5-ddgc). These zones were originally defined for administrative purposes by the NYC Department of City Planning. The rationale of this configuration is to provide a demand prediction for each of these administrative zones and make this result easier for city operators to understand. Figure 4 shows these taxi zones for the particular case of Manhattan.

#### 2.2.2. Required Datasets

In order to train the model for qualitative taxi demand prediction, three types of datasets are required, as explained next.

##### OSN Data

Three different OSN platforms are targeted by QUADRIVEN, namely Flickr (https://www.flickr.com), Foursquare (https://foursquare.com), and Brightkite (Brightkite is now a defunct OSN platform), with different purposes.

First of all, Foursquare and Brightkite are Location-based Social Networks (LBSNs). In this type of network, users check in at different places (venues) following a gaming interaction. Thus, each check-in post reflects that a particular user remained at a certain spatial location or venue during an undefined time interval. In addition to that, Flickr, the third source under consideration, is a photo-sharing platform. Hence, each time a user uploads a geo-tagged photo to Flickr, she/her also reveals her/his location around a certain point of interest. We believe that this location also represents the area where the user roamed during a certain period of time. Consequently, this work relies on the assumption that the three target OSNs are reliable sources to detect the presence of users in certain regions and time intervals.

For Flickr (FL), we extracted the user documents from the Flickr Creative Commons 100 M public repository [14]. In this way, we just kept the geo-tagged documents from the repository that fit into the spatial polygon defined for the Manhattan taxi zones (see Figure 4) covering a 22-month period (from January 2009 to October 2010). Regarding Foursquare (FS), we also used an open repository comprising worldwide check-ins from the platform (http://www.yongliu.org/datasets/). Accordingly, we also filtered the repository to only keep documents fitting into Manhattan taxi zone boundaries and covering the same 22-month period. As for Brightkite (BK), we used the open dataset available at the Stanford Network Analysis Project comprising 4,491,143 worldwide check-ins from the platform [15]. Similarly, we also filtered the dataset using the aforementioned taxi zones and time period.

**Figure 5:**
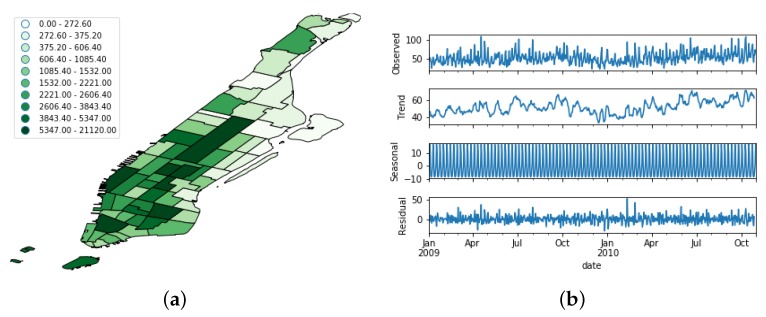
Distribution of the Flickr dataset. (**a**) Spatial distribution; (**b**) Temporal evolution. The uppermost figure shows the raw time series, whereas the other bottom ones depict its decomposition in trend, seasonal, and noise features.

**Figure 6:**
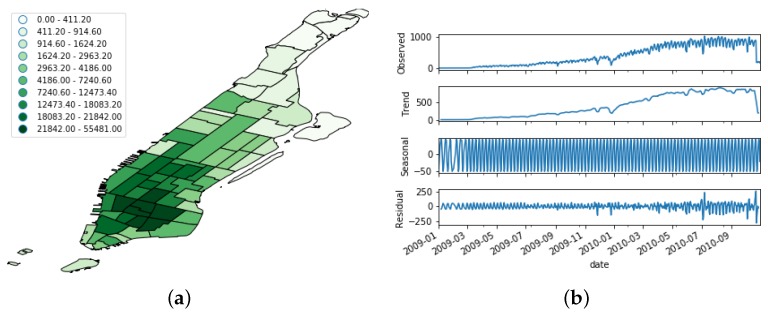
Distribution of the Foursquare dataset. (**a**) Spatial distribution; (**b**) Temporal evolution. The uppermost figure shows the raw time series, whereas the other bottom ones depict its decomposition in trend, seasonal, and noise features.

**Figure 7:**
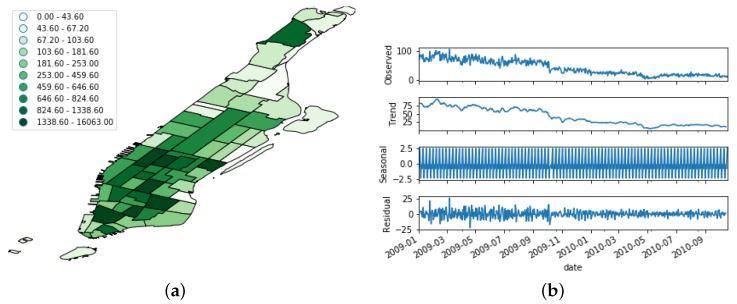
Distribution of the Brightkite dataset. (**a**) Spatial distribution; (**b**) Temporal evolution. The uppermost figure shows the raw time series, whereas the other bottom ones depict its decomposition in trend, seasonal, and noise features.

Figure 5, Figure 6 and Figure 7 show the spatial and temporal distribution of the documents on the three platforms, and Table 1 summarizes their details. Figure 5b, Figure 6b and Figure 7b show that the three OSN platforms followed very different temporal evolutions. While the number of active users in Flick remained more or less stationary during the whole time period, the number of Foursquare users followed a steady increment throughout the whole period of study. Besides, the evolution of Brightkite mirrored Foursquare as the number of active users decreased throughout the whole period under study. This behavior supports the hypothesis of the present work that OSN feeds are not stable data feeds, but time-variant ones.

In order to better understand this time-variant nature, we performed a seasonal decomposition using moving averages of the time series. This method decomposes the original time series into trend, seasonal, and residual factors by following an additive approach. As we can see from Figure 5b, Figure 6b and Figure 7b, the trend factor was the one that contributed most to the values composing the original time series. On the contrary, the seasonal dimension had a very narrow range of values in each of the three OSN platforms.

##### Original Taxi Demand Record Data

**Figure 8:**
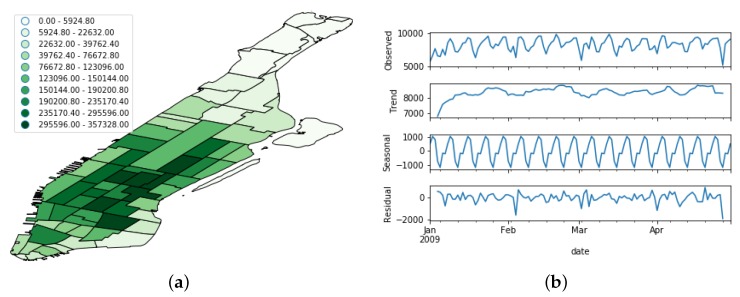
Distribution of the taxi demand dataset. (**a**) Spatial distribution; (**b**) Temporal evolution. The uppermost figure shows the raw time series, whereas the other bottom ones depict its decomposition in trend, seasonal, and noise features.

This dataset was extracted from the NYC Taxi and Limousine Commission Trip Record Data (https://www1.nyc.gov/site/tlc/about/tlc-trip-record-data.page). Each record in this dataset represents a particular taxi trip including pick-up and drop-off dates/times, pick-up and drop-off locations, trip distances, itemized fares, rate types, payment types, and driver-reported passenger counts. From the present setting, we crawled all the trip records whose pick-up location fit into any of the Manhattan taxi zones during the same 22-month time period used for the OSN data. As a result, 298,657,716 trip records were obtained, and only the pick-up date and location details of each trip were stored, discarding the rest of the trip features. Figure 8 shows the spatial distribution and temporal evolution of the resulting taxi demand record data.

##### Meteorological Data

This dataset was comprised of the weather conditions of Manhattan during the time period of study. It was extracted from an open web service hosting historical weather data from NYC (https://www.meteoblue.com/en/products/historyplus/download/new-york_united-states-of-america_5128581). Among the varied palette of available parameters, we eventually crawled the temperature and the rain level on an hourly basis.

### 2.3. Correlational Study

Table 2 shows the Normalized Mutual Information (NMI) score between the three OSN datasets and the taxi demand one. NMI allows capturing relations among datasets more complex than simple linear correlations [16]. To calculate this score, we aggregated the OSN documents and taxi pick-ups per taxi zone.

From this table, we can observe quite high NMI scores for the three OSN datasets under consideration with respect the taxi demand values. This indicates that there exists a strong relationship between the taxi demand behavior and the OSN activity in the different regions of the city.

### 2.4. Calculation of the Number of Active Users

As was stated in Section 2.1, QUADRIVEN takes as input the number of users uploading content in a particular spot and time period of the city ($U^{rh}$) to figure out the taxi demand prediction. However, this isolated parameter is not enough to come up with accurate predictions. Instead, we need to infer whether the values at $U^{rh}$ actually represent a high or low number of users for such an area and time period. For that goal, QUADRIVEN takes a batch processing approach. By means of a time based sliding window, we kept the OSN user count data for the last *p* days. Let us define ${\mathrm{OU}}_{d}^{rh}=\langle {\langle ou{\left(d\right)}_{i}^{rh},ou{\left(d-1\right)}_{i}^{rh},\ldots ,ou{\left(d-p-1\right)}_{i}^{rh}\rangle }_{\forall i\in \langle FS,FL,BK\rangle }\rangle$ as the set comprising such historical data where $ou{\left(d\right)}_{i}^{rh}$ is the number of users that uploaded content in region *r* at hour *h* during day *d* on the OSN *i*.

As the sliding windows moves and a new batch sample ${\mathrm{OU}}_{d^{\prime }}^{rh}$ is generated, we calculate the sampling distribution of the mean ${\bar{X}}_{i}^{rh}$ of the OSN *i* in region *r* at hour *h* during the last *p* days. This distribution follows a normal distribution ${\bar{X}}_{i}^{rh}∼N\left(\mu_{i},\sigma_{i}^{2}/p\right)$ when *p* > 30 [17] where $\mu_{i}$ is the sampling mean and $\sigma_{i}^{2}$ is the sampling variance.

Given the aforementioned distributions ${\bar{X}}_{i}^{rh}\forall i\in \left[FS,FL,BK\right]$, we can now re-formulate the count values in $U^{rh}$ as a z-score set $Z^{rh}=\langle z_{FS}^{rh},z_{FL}^{rh},z_{BK}^{rh}\rangle$ where,

$$z_{i}^{rh}=\frac{u_{i}^{rh}-\mu_{i}}{\sigma_{i}/p}$$

As a result, the new set $Z^{rh}$ not only normalizes the count values of the original set $U^{rh}$, but also, its z-score values actually represent how high or low the original number of active users is with respect to the average number in a particular region and hour.

#### Home User Filtering

One of the flaws that occurs when counting the total number of active OSN users in a certain area is that we also include users whose home location is inside the area under consideration. However, these residents are not really interesting in terms of taxi demand. On the contrary, we should focus on the floating population in the area. For that reason, we removed from each sliding dataset $U^{rh}$ all users whose home location was within the target region *r*. To do so, we searched for the most frequent location of each user at night hours as this is a commonly accepted approach in the mobility mining literature for home location identification [18]. Then, we removed from $U^{rh}$ all users whose estimated home location was inside *r*.

**Figure 9:**
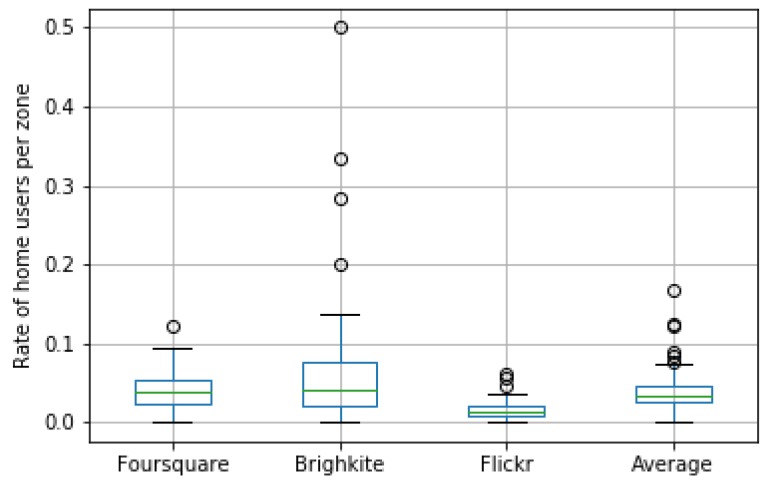
Rate of home users per OSN and zone along with the averaged rates comprising all the OSNs.

**Figure 10:**
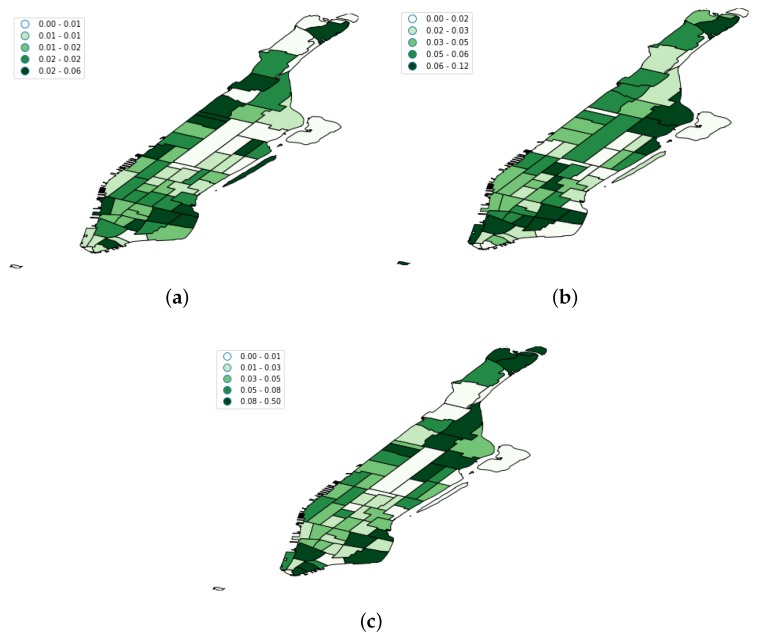
Spatial distribution of home user locations per taxi zone in the three OSNs. (**a**) Foursquare home user rates; (**b**) Flickr home user rates; (**c**) Brightkite home user rates.

Figure 9 shows the results of this filtering process when it was applied to the OSN datasets. We can see that, on average, 5% of the total users were removed. However, there were meaningful differences among platforms according to Figure 9. Whilst the variability of the removed users was quite large for Brightkite, Flickr exhibited a much more flat behavior. This is compatible with the nature of each OSN platform. Flickr is a picture-based social network that is frequently used by tourists when they go sightseeing, whereas Brightkite and Foursquare users usually check in at venues that take part of their frequent spots. This justifies the low rate of residents found on Flickr. Lastly, Figure 10 shows the spatial distribution of the removed home users per taxi zone.

For the sake of clarity, Algorithm 1 sums up all the aforementioned pre-processing stages to prepare the input OSN data for further processing.

**Algorithm 1:** Pseudo-code of the Z-scores calculation for OSN count data, including home-user data removal.

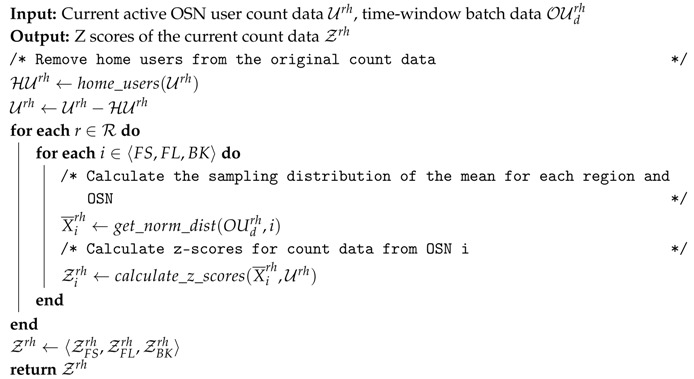



### 2.5. Calculation of the Taxi Demand Quantiles

**Figure 11:**
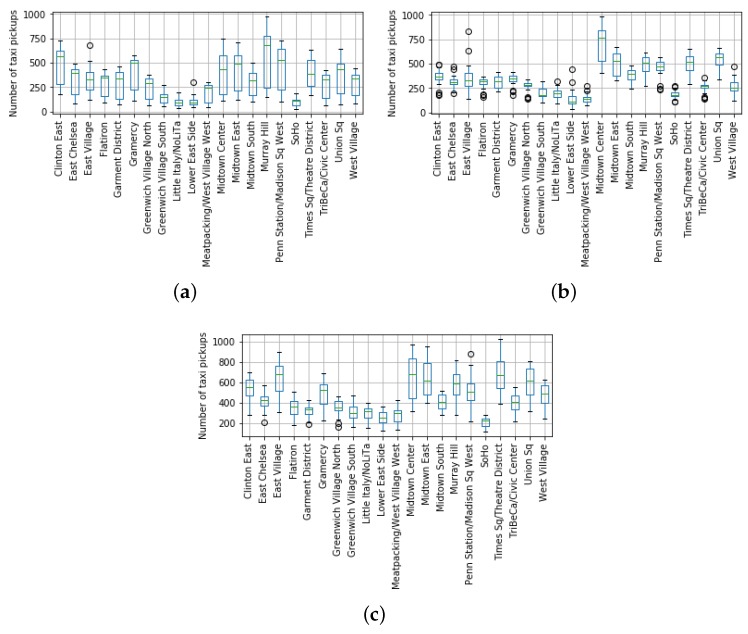
Boxplots of the pick-ups per taxi zone at three particular hours of the day. The top side of each box represents the upper quantile $Q_{3}^{rh}$ of a region, whereas the bottom side stands for the lower one $Q_{1}^{rh}$. (**a**) 8:00; (**b**) 15:00; (**c**) 21:00.

Regarding the original taxi demand data (see Section 2.2.2), we needed to translate its numerical records into a categorical dataset. To do so, the following procedure was performed:

- Firstly, we aggregated the records per taxi zone and hour for each of the dates in the 22-month period. Let us define $tp_{d}^{rh}$ as the number of taxi pick-ups at region *r* at hour *h* in day *d*.
- Next, we created a set comprising all the $tp_{d}^{rh}$ values for every region and hour. This gave rise to r×h stratified sets ${\mathrm{TP}}^{rh}$. For example, the set ${\mathrm{TP}}^{4,9}$ comprised all the count values $tp_{d}^{4,9}$ with the number of pick-ups at Region #4 at 9:00 a.m. for all the dates *d* of the original dataset.
- Then, we calculated the lower ($Q_{1}^{rh}$) and upper quartiles ($Q_{3}^{rh}$) for each ${\mathrm{TP}}^{rh}$ set. At this point, we should remark that these quartiles were calculated for each particular region at a single hour of the day. This is because the taxi demand profile meaningfully varied depending on the target region and the hour of the day, as Figure 11 shows. This way, the obtained quartiles actually represent low and high boundaries of the taxi demand behavior in a region regardless of seasonal patterns.
- Finally, we mapped each $tp_{d}^{rh}$ value to their corresponding quartile range (low, middle, high) defined as $\langle tr_{low}^{hr},tr_{middle}^{hr},tr_{high}^{hr}\rangle$ by means of the following if-then rules,
  – If $tp_{d}^{rh}\leq Q_{1}^{rh}$, then the assigned label is $tr_{low}^{hr}$.
  – If $tp_{d}^{rh}>Q_{1}^{rh}\;\mathrm{and}\;tp_{d}^{rh}\leq Q_{3}^{rh}$, then the assigned label is $tr_{middle}^{hr}$.
  – If $tp_{d}^{rh}>Q_{3}^{rh}$, then the assigned label is $tr_{high}^{hr}$.

All in all, this procedure assigned a categorical label (low, middle, or high) to a taxi demand count value $tp_{d}^{rh}$ that represented how important such demand was with respect to the demand profiling of the zone and hour of the day. Figure 11 shows that such demand profiling meaningfully varied not only among zones, but also through hours.

As a matter of fact, according to Figure 11 and the aforementioned if-then rules, a taxi demand of 500 pick-ups at Clinton East zone (the leftmost zone in the x-axis of plots in Figure 11) would be labeled as middle at 8:00 and 21:00 because this value was between the lower and upper quartiles for both hours. However, the same value would be labeled as high at 15:00 as it was above the upper quantile of this zone and hour ($Q_{3}^{ClintonEast-3pm}$).

### 2.6. Composition of the Classifier

Bearing in mind the two procedures explained above for z-score calculation from OSN data and quartile-based labels for taxi records, the input and outputs of the final model are now defined to carry out with the prediction task. The taxi demand prediction is defined as a classification problem, and therefore, the final model proposed here is a classifier. The classifier takes as independent variables the following features:

- the target region *r*,
- the current hour of the day *h*,
- the current day of the week $d_{week}\in \langle 0,\ldots ,6\rangle$,
- The z-scores $z_{FS}^{rh},z_{FL}^{rh},z_{BK}^{rh}$ of the three OSNs for region *r* at hour *h*,
- the current temperature *t*,
- the current rain level rl.

With all these input variables, the classifier generated one of the three labels related to the qualitative estimated demand in the region *r* at hour h+1. Hence, the classifier is defined as a function as follows,

$$f:\langle r,h,d_{week},z_{FS}^{rh},z_{FL}^{rh},z_{BK}^{rh},t,rl\rangle \to \langle tr_{low}^{(h+1)r},tr_{middle}^{(h+1)r},tr_{high}^{(h+1)r}\rangle$$

All in all, the classifier did not rely on any taxi records as part of its input vector to perform its prediction, but on temporal (*h*, *d*), meteorological (*t*, rl), and OSN normalized data ($z_{FS}^{rh},z_{FL}^{rh},z_{BK}^{rh}$) to generate a taxi demand estimation category that took into account the taxi demand profiling of the target region.

## 3. Evaluation of the Proposal

This section performs an evaluation of the suitability of QUADRIVEN by instantiating different classification models.

### 3.1. Evaluated Models

Five different techniques coming from the supervised machine learning field were used to provide a comprehensive evaluation. Each one follows a completely different approach to solve a classification problem.

#### 3.1.1. Conditional Random Fields

A CRF is a probabilistic graphical model that has been widely applied in the natural language processing and object recognition fields [19]. Furthermore, CRFs have been also utilized in the mobility mining field for location prediction [20]. In brief, CRF maps an observation sequence X to a sequence of labels Y. Then, we can use it to estimate the conditional probability of Y given an observation sequence X.

In our setting, we fed a CRF with the sequence of input features during the last eight hours (〈h,h−1,…,h−7〉) in a region *r* to generate the prediction for hour h+1. The rationale of using this type of graphical model was that taxi demand in a region *r* at a particular hour *h* might be influenced not only by the human activity at the previous hour, but also during a certain previous period of time.

#### 3.1.2. Random Forest

This popular supervised algorithm takes the form of an ensemble of decision trees combining the predictors. A decision tree is just a tree-based graph comprising a set of decision nodes that evaluates particular features of the input vector. The leaf nodes are labeled with the values of the dependent variable. The classification of particular instance is done by trespassing the trees from the root to a particular leaf node.

The reason to choose this model is twofold. Firstly, it is one of the most successfully applied algorithms to perform classification tasks based on tabular data [21]. Secondly, its tree-based nature allows composing explanatory models.

#### 3.1.3. Support Vector Machine

SVM is one of the foremost machine learning algorithms for classification tasks. In brief, an SVM focuses on finding a hyperplane to separate the samples in the dataset properly by maximizing the distance between itself and the samples. In this context, SVMs have been successfully applied to classify spatio-temporal data in urban domains [22]. Besides, by including this model among the set of candidates, we were able to study the suitability of using kernel based algorithms with our framework.

#### 3.1.4. Long Short Term Memory Neural Network

LSTM is a particular type of Deep Neural Network (DNN) that can handle long term dependencies due to a specially crafted memory cell. This way, the LSTM approach is able to forget and select information using dedicated neural networks. As a result, they have been widely used to analyze time series for regression and classification tasks. By considering LSTMs in this evaluation, we provide an alternative to the aforementioned CRF model to capture long term dependencies in human activity that might affect the taxi demand estimation.

#### 3.1.5. Fully Connected Neural Network

A Fully Connected Neural Network (FCNNs) is a basic type of DNN. It basically comprises a set of fully connected layers of neurons where neurons receive an input, perform some operation on it, and forward the result to neurons on the upcoming layers. DNNs have been widely used in the taxi demand prediction problem with very accurate results when they are used as a regression model (see Section 4). Consequently, we studied this algorithm so as to evaluate its suitability for our classification point of view.

### 3.2. Implementation Details

This evaluation was implemented using Python 3.6 as the programming language with scikit-learn [23] as the orchestration framework. In addition to that, the CRF model was generated using the CRF-suite library (https://python-crfsuite.readthedocs.io/en/latest/), RF, and SVM with scikit-learn and FCNN and LSTM by means of the Keras framework (https://keras.io).

### 3.3. Evaluation Settings

In order to evaluate our proposal, we set the first 20 months (January 2009 to August 2010) of the target time period as the training dataset and the remaining two months (September 2010 to October 2010) as the evaluation period.

Table 3 shows the particular configuration for each of the five models under consideration. To obtain such configurations, each model was executed several times with different configurations and training datasets in order to find our optimal parameter settings by following a three-fold cross-validation approach.

### 3.4. Evaluation Metrics

In order to evaluate the classifiers, we used the F1 score as the measurement. This score is calculated following the present formula:

$$F1=2\times \frac{precision\times recall}{precision+recall}$$

where:

$$recall=\frac{True\;positives}{True\;positives+False\;negatives}$$

$$precision=\frac{True\;positives}{True\;positives+False\;positives}$$

### 3.5. Results’ Discussion

**Figure 12:**
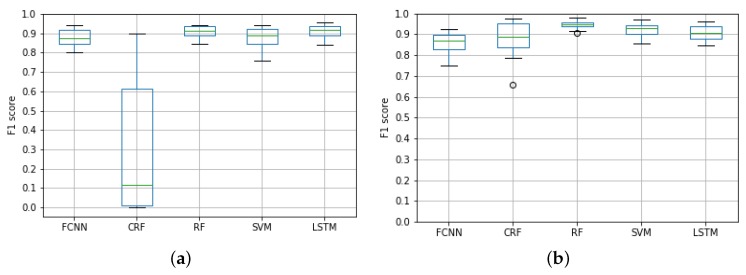
Average F1 score per zone and hour of each of the evaluated models considering both the OSN data and the taxi demand as input. (**a**) F1 score of QUADRIVEN models; (**b**) F1 score using taxi demand data.

Figure 12a shows the average F1 score of the five models considering all the hours and taxi regions. Besides, Figure 12b depicts the F1 score for the same algorithms, but this time, they took the real taxi demand as the primary input source. This way, these models replaced the $\langle z_{FS}^{rh},z_{FL}^{rh},z_{BK}^{rh}\rangle$ input variables of the original QUADRIVEN models (see Section 2.6) by the number of taxi pick-ups at hour *h*.

Unsurprisingly, the results showed that models directly fed with the actual taxi demand values were able to achieve a slightly higher classification accuracy than their OSN-based counterparts. This is especially noticeable for the CRF model. However, in general terms, the OSN-based solutions achieved F1 scores above 0.8, which were comparable with the ones achieved by using the real taxi demand. This similarity was due to the aforementioned high information similarity between the two data sources, as stated in Section 2.3.

Focusing on the QUADRIVEN models, CRF achieved a lower F1 score with the mean around 0.2. It is true that the model exhibited a variability higher than the other four algorithms. This might be due to the fact that the human activity during the last eight hours is only relevant at certain regions and hours.

**Figure 13:**
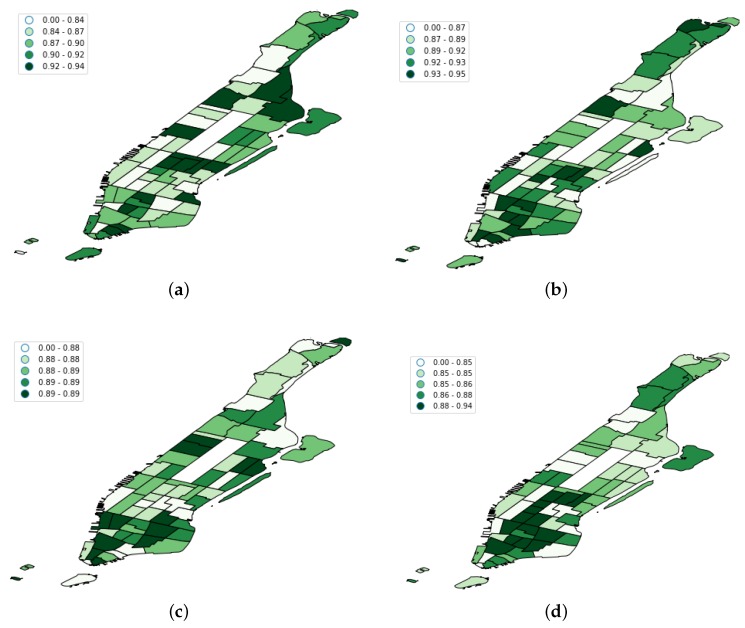

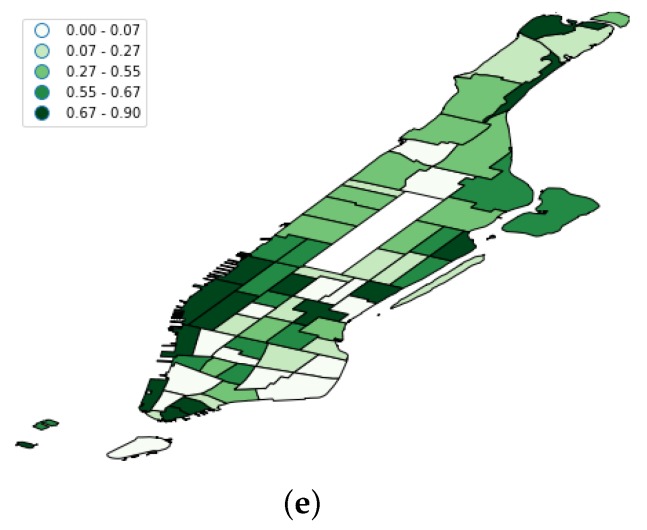
F1 score per taxi zone for each of the evaluated models. (**a**) FCNN F1 score per taxi zone; (**b**) LSTM F1 score per taxi zone; (**c**) RF F1 score per taxi zone; (**d**) SVM F1 score per taxi zone; (**e**) CRF F1 score per taxi zone.

To analyze the variability of the models with respect to the spatial distribution, Figure 13 shows the F1 score of each model per taxi zone. In that sense, we can see that, in general terms, the taxi zones at the south of Manhattan (the leftmost ones at the figures) were generally the regions with the highest F1 scores. This was because these regions contain some of the most popular tourist attractions of the borough (e.g., World Trade Center, the 09-11 Memorial, or the boat lines to visit the Statue of Liberty). This caused the presence of a high number of floating population in these areas.

Another interesting finding was that the CRF model achieved better results in the southwest regions (see Figure 13b). According to the official New York City planning website (https://zola.planning.nyc.gov/about#9.72/40.7125/-73.733), these taxi regions mainly cover districts for commercial purposes and high density residential areas like Tribeca or Chelsea Market. Therefore, taxi demand in such commercial and residential areas seemed to be affected not only by the most recent human activity, but also by human flows at certain hours in the past.

**Figure 14:**
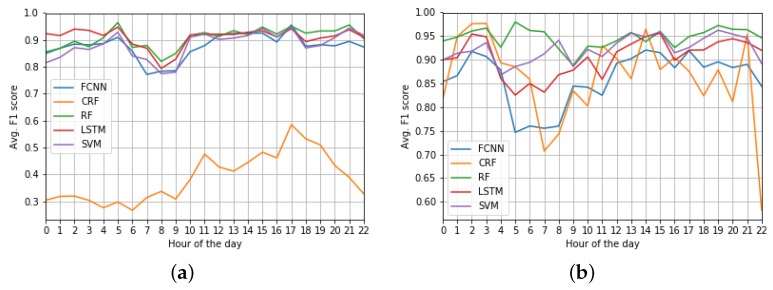
Average F1 score per hour of the day of each of the evaluated models considering both OSN and taxi demand data as input. (**a**) QUADRIVEN models; (**b**) Taxi-demand models.

In addition to that, we also studied the accuracy of the models with respect to the hour of the day, as Figure 14 shows. Again, this figure includes the scores of the QUADRIVEN models and the ones from the models directly fed with taxi demand data, as in Figure 12.

In comparative terms, we can see that the QUADRIVEN models followed a more homogeneous behavior than their taxi demand counterparts. This is especially remarkable in the case of the CRF model. Whilst the F1 score of the QUADRIVEN version followed a slight increase until 17:00 (see Figure 14a), the taxi data version exhibited several peaks and drops in its accuracy depending on the hour of the day (see Figure 14b). Furthermore, results in Figure 12b and Figure 14b confirm that the RF model was the most accurate solution among the five taxi demand fed candidates.

Focusing on the QUADRIVEN models, we can make some interesting remarks. First of all, the RF, SVM, LSTM, and FCNN models obtained their lowest F1 scores at the early hours in the morning (6.00–9:00). Since this time period comprises the taxi demand’s peak hours in the morning, this accuracy drop might be due to the fact that the algorithms were not able to capture the complex relations between OSN z-scores and taxi demand quartiles that occur in such a time period.

On the contrary, the CRF model achieved better results at 18:00. This is compatible with the aforementioned findings related to the spatial distribution of the F1 score of the model (see Figure 13b). In that sense, this hour is when most shops and other venues in commercial areas usually close, and thus, clients need some means of transport to leave them. Therefore, the actual taxi demand at such an exit hour relies on the amount of people that had entered these areas during the last hours and now need to got out at the same time. Due to its nature, the CRF model is able to capture this long standing behavior.

Finally, Table 4 contains the normalized confusion matrix of the models. From this matrix, we can see that LSTM and RF achieved quite high rates in the three target labels with values above 0.75, whereas CRF obtained much more inaccurate results. For example, RF was able to predict 76.3% of the high taxi demand ranges ($tr_{high}$) correctly, whereas LSTM predicted 77% of these labels.

Moreover, LSTM did not misclassify high taxi demand ranges as low ranges, whereas RF only did so in 1% of the labels. Avoiding this type of error is critical, as anticipating a peak in taxi demand in which a drop would actually occur would have a serious impact on the taxi operators. Similarly, LSTM almost did not misclassify low taxi ranges as high ones (0.2%), and RF only did so in 1% of the cases.

All in all, in light of all the aforementioned results, we can conclude that LSTM seems the most suitable algorithm to instantiate QUADRIVEN. It did not only achieve the highest F1 score among competitors, but also the most stable one in terms of time evolution and confusion matrix values.

### 3.6. OSN Data Sources Comparison

**Figure 15:**
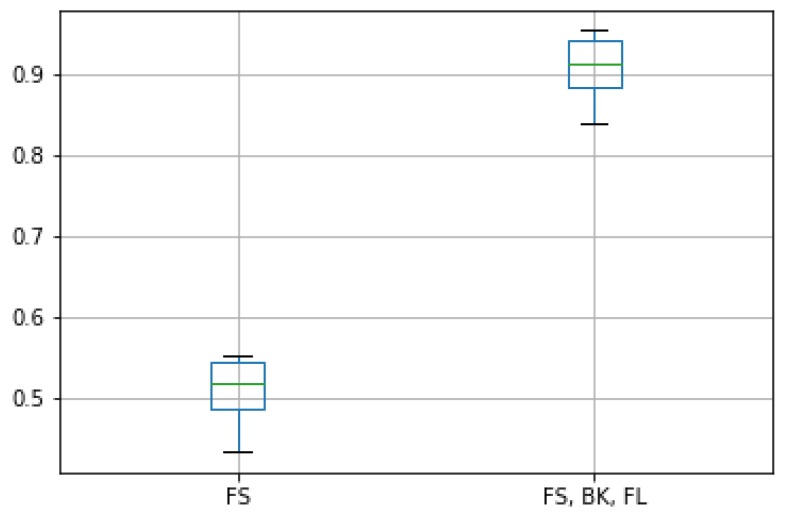
F1 score of the original LSTM model integrating the three OSN platforms and the alternative version considering only FS as OSN data.

Finally, we studied the effect of using only one out of the three OSN platforms as input data. The goal of this experiment was to evaluate the suitability of using three different OSN platforms as primary input sources. For that goal, we trained an alternative version of the LSTM model that only took the z-scores of the Foursquare (FS) platform. Figure 15 shows the F1 scores of this alternative LSTM version along with the original one.

We can see that the accuracy of the model meaningfully decreased when a single OSN source was used. The rationale for this drop was that the z-score from an isolated OSN source was not able to capture the rich variations of human presence in the target urban areas. On the contrary, using three different z-scores increased the dimensionality of the input. Hence, we were able to separate more clearly these variations to associate them with the resulting taxi demand. Furthermore, since the z-scores were defined in the same range of values ([0,1]), the three OSNs contributed equally to this detection.

## 4. Related Work

When it comes to predicting the demand of mobility services (e.g., taxis, bike-sharing, car-sharing, etc.) with data mining techniques, three different characteristics can be established to catalog the existing literature: the type(s) of incoming data, the method to perform the prediction, and the type of demand prediction generated (see Table 5). A review on each of these characteristics is given next.

### 4.1. Input Data Sources

Regarding the input sources used for taxi-ride inference, there is a varied range of alternatives. However, they can be summed up as four different categories. As Table 5 shows, most of the approaches for taxi demand prediction have made use of several temporal (e.g., time of the day, hour, month, and the like), spatial (e.g., land use data), and meteorological (e.g., temperature) features of the urban environment. This is because such factors have proven to have a meaningful impact on the behavior of the taxi users [2].

Concerning the primary feeds to infer taxi-ride information actually, a prominent course of action has focused on using historical taxi demand information in a quantitative manner. These data usually take the form of pick-up and drop-off records registered by the taxi fleet of the target city. For example, the work in [24] made use of the taxi demand during the last *n* days for its analysis. Similarly, the work in [29] counted the number of pick-ups in gridded cells, whereas the work in [6] also considered drop-offs and compared them with baseline values as part of the input data. In [13,25], taxi demand was modeled as square images where each pixel represented an area under consideration. Moreover, the work in [12] considered taxi demand records as time series and extracted features like closeness trend and period. In [9,27], taxi records were used to compose an origin-destination matrix representing the passenger flows within the city of interest.

An interesting approach was provided in [7], where two types of data sources were fused, Call Detail Records (CDRs) from cell-phone networks and real-time taxi demand records. In such a solution, CDRs were used to estimate the total population in the areas of interest. The work in [26] also relied on different primary sources to improve the taxi prediction in two particular spots of a city. In this case, taxi pick-up and drop-off records were enriched with information about events (e.g., concerts, exhibitions, and the like) happening in the two particular urban areas monitored by the proposal.

Social media data have been scarcely used in the taxi demand prediction problem. An illustrative example can be found in [8], which studied the feasibility of Twitter as a primary source for this goal. Besides, the work in [2] enriched the taxi demand records with check-in data from OSN platforms to identify region’s venue distributions.

Finally, taxi GPS traces have also been used for some works, as they provide the whole path followed by taxicabs during their rides [3,28]. These traces frequently contains other data such as the timestamp, the status of the taxi (i.e., occupied or not), etc.

QUADRIVEN only takes as primary input data the social media documents published on OSN platforms during their operational stage, as put forward in Section 2.2.2. This data source has not been fully exploited by existing taxi demand predictors. Unlike other approaches relying on social media data [2,8], our solution uses multiple OSN feeds instead of a single one. Moreover, we actually estimated the number of potential taxi passengers in a urban area instead of just for land-use profiling, like in [2]. Finally, as put forward in Section 2.4, our solution was able to adapt itself to the time-variant nature of OSN feeds instead of providing a monolithic solution, like in [8].

### 4.2. Data Mining Methods

When it comes to analyzing the aforementioned data sources, a dominant course of action to extract meaningful knowledge has focused on different regression techniques. In this way, the work in [24] proposed a Linear Regression (LR) model with high-dimensional features. Similarly, Decision Trees (DTs) have also been used as a prediction method [2,8] along with Least Squares Support Vector Machines (LS-SVM) [29]. A linear regression model combined with a Gaussian process (GP) was proposed in [26]. A comparison of other regression methods, including ARIMA and a time-varying Poisson model, was explored in [28] to predict the taxi demand, the ensemble learning being the one showing more accurate results.

Neural Networks (NN) have been a recurrent technique in the taxi-data mining field. As a matter of fact, the work in [3] applied a fine-tuned Multi-Layer Perceptron (MLP) in their solution and compared it with other NN techniques like Recurrent Neural Networks (RNN) or memory networks. The work in [13] introduced a multi-NN approach combining Convolutional Neural Networks (CNNs) and Long Short Term Memory Networks (LSTN) for analytic purposes. Similarly, an Autoencoder method (AE) was used in [7], and the work in [6] made use of CNNs as the underlying machine learning method. Besides, the work in [9] made use of a convolutional long short-term memory network to integrate the outcome of different contextual modules. In [25], a CNN was also used, but in this case, individual and region based fairness methods were incorporated. A complex approach fusing a 3D CNN and an LSTN was introduced in [12]. Whereas the former dealt with pick-up records, the latter embedded other spatio-temporal features. In some cases, NNs were just used to embed certain features, so as to be further analyzed by other models like Support Vector Regression (SVR) [27].

In this context, our work states that the taxi demand prediction problem can be regarded as a classification task instead of a regression one. This view makes it possible to explore alternative solutions for this problem based on the large variety of classification models within the data mining field, as has been evaluated in Section 3.1.

### 4.3. Prediction Outcome

Finally, we can distinguish three types of predictions provided by the previously described data mining methods. To begin with, existing works usually focus on inferring the future taxi demand in certain urban areas in a quantitative manner, that is the estimated number of users that will ask for a taxi in the next hours [2,7,13,24,25,26,28,29]. In most of the cases, the prediction covers the demand in a large portion of the target city [2,7,12,13,24,25,28,29], but in some cases, solutions just focus on particular spots like mobility hubs or convention centers [26]. One interesting approach was presented in [9], where the taxi demand forecasting was modeled as origin-destination matrices.

Other works tries not to predict a continuous taxi demand flow, but particular demand events instead. A clear example of this is the forecasting of taxi demand peaks, that is time periods covering abnormally high taxi demands. In this context, the work in [6] detected such peaks by means of survival analysis.

Finally, another prominent goal has been the destination prediction of taxi rides so as to anticipate the particular drop-off point of a taxicab [3].

Unlike previous solutions, QUADRIVEN provides taxi demand forecasting using quantile-based categories, as described in Section 2.5. This way, each category represents an estimated demand level covering low, average, and high demand situations. In that sense, it is true that the aforementioned quantitative predictions based on an estimated number of taxi pick-ups could be also converted into quantile-based outcomes by appending an extra indexation level. However, the underlying models that generate the original numerical predictions do not consider the quantile ranges in their prediction process as our solution does.

## 5. Conclusions

The operational optimization of public means of transport in cities is an important action so as to reduce the environmental impact and develop more sustainable cities. In that sense, taxicabs are one of the most widespread and popular mobility services in urban areas. For that reason, several works already exist to anticipate taxi demand behaviors and, thus, achieve the aforementioned operational optimization.

In this context, the work at hand put forward QUADRIVEN, a novel approach for taxi demand prediction based on a classification point of view. This way, a set of labels defining different taxi demand ranges was defined as the potential outcome of the classifier. This allowed anticipating peaks, drops, and regular taxi demand behaviors. As predictors, the solution took normalized OSN count data from several platforms to sense the human presence in a set of pre-defined spatial regions. Lastly, we tested our approach in Manhattan by instantiating three algorithms, each one following a different paradigm. Results showed quite high classification rates in at least two of the models.

Finally, future work will focus on integrating new input variables like traffic event information, spatio-temporal trajectories extracted from OSN users, or the actual demand for other mobility services (e.g., metro, bus, and the like). We are also considering the inclusion of the analysis of the actual post content associated with the geolocation data and the study of the user mobility patterns. In this sense, the leverage of OSNs different from the specific location-based ones, such as Twitter, will be worth exploring. All these sources of information would allow us to capture a more holistic view of the contextual mobility of an urban area and improve the classification capabilities of the framework.

## Figures and Tables

**Table 1:** OSN raw dataset details during the target time period of January 2009 to October 2010.

	Flickr	Foursquare	Brightkite
Number of users	5576	4531	2630
Number of documents	244,464	628,941	70,642

**Table 2:** Normalized mutual information score between the OSN dataset and the taxi demand.

OSN	Taxi Demand
Flickr	0.9895
Foursquare	0.9871
Brightkite	0.9749

**Table 3:** Model parameters.

Model	Parameter	Value
CRF	Training algorithm	Gradient descent
	L1 regularization coeff.	0.1
	L2 regularization coeff.	0.1
	Max. iterations	1000
RF	Number of estimators	12,000
	Max. deep	1100
SVM	Kernel	Radial Basis Function (RBF)
	Gamma	0.001
	C	1000
FCNN	Number of layers	8
	Number neurons per layer	128
	Activation function	ReLU
LSTM	Number of layers	3
	Number neurons per layer	50
	Activation function	ReLU

**Table 4:** Confusion matrix of the five models under study. The best rates per model are marked in bold. The grayed cells contain the highest rates per true label.

Model	Predicted Taxi Range	True Taxi Range		
		${\mathrm{tr}}_{\mathrm{high}}$	${\mathrm{tr}}_{\mathrm{middle}}$	${\mathrm{tr}}_{\mathrm{low}}$
RF	$tr_{high}$	**0.763**	0.232	0.005
	$tr_{middle}$	0.114	**0.768**	0.117
	$tr_{low}$	0.010	0.207	**0.783**
SVM	$tr_{high}$	**0.717**	0.270	0.013
	$tr_{middle}$	0.059	**0.792**	0.149
	$tr_{low}$	0.100	0.161	**0.829**
FCNN	$tr_{high}$	**0.701**	0.293	0.006
	$tr_{middle}$	0.088	**0.797**	0.115
	$tr_{low}$	0.011	0.248	**0.741**
LSTM	$tr_{high}$	**0.770**	0.228	0.002
	$tr_{middle}$	0.059	**0.840**	0.100
	$tr_{low}$	0.000	0.152	**0.846**
CRF	$tr_{high}$	**0.561**	0.435	0.004
	$tr_{middle}$	**0.627**	0.344	0.028
	$tr_{low}$	**0.554**	0.281	0.165

**Table 5:** Key features of existing prediction models for mobility services. The acronyms’ meaning is as follows: LR, Logistic Regression; MLP, Multi-Layer Perceptron; CNN, Convolutional Neural Network; LSTM, Long Short Term Memory Networks; AE, Auto Encoder; DT, Decision Trees; GP, Gaussian Process and SVR, Support Vector Regression; EL, Ensemble Learning; ConvLSTM, Convolutional Long Short Term Memory Network; LS-SVM, Least Squares Support Vector Machine.

Reference	Data Sources				Data Mining Method	Prediction Target
	Temporal	Spatial	Meteorological	Primary Input Data		
[24]	✓	✓	✓	taxi demand	regression/LR	quantitative taxi demand
[3]	✓			taxi GPS traces	regression/MLP	taxi destination
[13]	✓	✓	✓	taxi demand	regression/CNN, LSTM	quantitative taxi demand
[7]	✓		✓	taxi demand and CDRs	regression/AE	quantitative taxi demand
[6]	✓		✓	taxi demand	regression/CNN	taxi demand peaks
[25]	✓	✓	✓	bike demand	regression/CNN	quantitative bike demand
[2]	✓		✓	taxi demand and social media	regression/DT	quantitative taxi demand
[26]				taxi demand and event data	regression/LR, GP	quantitative taxi demand
[12]	✓	✓	✓	taxi demand	regression/CNN, LSTM	quantitative taxi demand
[27]	✓			taxi demand	regression/SVR	quantitative taxi demand
[8]	✓			taxi demand and social media	regression/DT	quantitative taxi demand
[28]	✓	✓		taxi GPS traces	regression/EL	quantitative taxi demand
[9]	✓	✓	✓	taxi demand	regression/ConvLSTM	quantitative taxi demand
[29]	✓			taxi demand	regression/LS-SVM	quantitative taxi demand
QUADRIVEN	✓		✓	social media	classification	qualitative taxi demand
